# Seasonal variations in pancreatic surgery outcome *A *retrospective time-trend analysis of 2748 Whipple procedures

**DOI:** 10.1007/s13304-020-00868-6

**Published:** 2020-08-20

**Authors:** Giovanni Marchegiani, Stefano Andrianello, Chiara Nessi, Tommaso Giuliani, Giuseppe Malleo, Salvatore Paiella, Roberto Salvia, Claudio Bassi

**Affiliations:** Department of General and Pancreatic Surgery, The Pancreas Institute, University of Verona Hospital Trust, P.le Scuro 10, 37134 Verona, Italy

**Keywords:** Seasonal variations, Outcomes, Surgery, Pancreas, July effect

## Abstract

**Background:**

Observing cyclic patterns in surgical outcome is a common experience. We aimed to measure this phenomenon and to hypothesize possible causes using the experience of a high-volume pancreatic surgery department.

**Methods:**

Outcomes of 2748 patients who underwent a Whipple procedure at a single high-volume center from January 2000 to December 2018 were retrospectively analyzed. Three different hypotheses were tested: the effect of climate changes, the “July effect” and the effect of vacations.

**Results:**

Clavien-Dindo ≥ 3 morbidity was similar during warm vs. cold months (22.5% vs. 19.8%, *p* = 0.104) and at the beginning of activity of new trainees vs. the rest of the year (23.5 vs. 22.5%, *p *= 0.757). Patients operated when a high percentage of staff is on vacation showed an increased Clavien-Dindo ≥ 3 morbidity (22.3 vs. 18.5%, *p* = 0.022), but similar mortality (2.3 vs. 1.8%, *p* = 0.553). The surgical waiting list was also significantly longer during these periods (37 vs. 27 days, *p* = 0.037). Being operated in such a period of the year was an independent predictor of severe morbidity (OR 1.271, CI 95% 1.086–1.638, *p* = 0.031).

**Conclusion:**

Being operated when more staff is on vacation significantly affects severe morbidity rate. Future healthcare system policies should prevent the relative shortage of resources during these periods.

## Introduction

Institutional experience, team cohesion and personal skills are crucial to achieving excellence in surgery. Excellence means better outcomes, such as those obtained through the centralization of high-risk surgical procedures at high-volume hospitals [1]. These facilities have a broader range of specialists and technology-based services, different types of intensive care units, more resources and highly standardized clinical pathways that can provide the complex perioperative care needed for patients undergoing major surgical procedures.

Despite such a high level of standardization of care, a seasonal variability in outcomes is a common experience even if an evidence-based approach has led to variable results [2–8]. Seasonal climate changes have been identified as the possible cause of worse surgical outcomes during the summer months, especially for surgical site infections [9]. Other studies have reported an increased morbidity rate in July and August at the beginning of the academic year, when new trainees and residents provide patient care for the first time [2–4]. The so-called July effect would therefore be related to the negative influx of relatively inexperienced trainees who are unfamiliar with their roles and responsibilities. Another pattern characterized by outcome deterioration has been linked to sabbaticals. Several studies have reported increased morbidity and worse survival outcomes after major oncological procedures performed on Fridays [10], during the weekend [11] or on holidays [12] due to the shortage of medical and nursing staff.

However, the heterogeneity of surgical procedures and the lack of standardization on outcome metrics [13] has prevented the drawing of more precise inferences in this field.

Major pancreatic resections are complex but highly standardized procedures with specific outcome metrics [13] that are usually centralized in large, high-volume academic centers. These features make pancreatic surgery an ideal model for exploring the seasonal variability in surgical outcomes.

The aim of the present paper is to assess the presence of specific patterns in surgical outcome variability, to measure this phenomenon and to try to explain possible causes.

## Methods

All Whipple procedures consecutively performed for all indications from January 2000 to December 2018 at the Department of General and Pancreatic Surgery—The Pancreas Institute, University of Verona Hospital Trust, were identified from a prospectively maintained institutional database. Only elective procedures were included.

The study was approved by the Institutional Review Board (approval number: 1101CESC, informed consent waived) and followed the statements developed by the “Strengthening the Reporting of Observational Studies in Epidemiology” (STROBE) guidelines.

Short-term outcomes were registered, as well as baseline clinical, intraoperative and pathological variables. All procedures were performed by a team of surgeons composed by two senior pancreatic surgeons and two residents. When feasible and safe, a senior resident (post-graduate year 5 or 6) carried out the Whipple procedure under the constant supervision of a senior surgeon.

The surgical technique has remained essentially unchanged, but it has been influenced by the technological evolution of surgical devices [14]. Other important milestones are represented by the increasing use of neoadjuvant therapy since 2015 [15, 16], postoperative pancreatic fistula (POPF) risk stratification introduced in 2014 [17] as well as the implementation of enhanced recovery after surgery pathways [18]. The pancreatic anastomoses performed were dunking pancreaticojejunostomy (International Study Group for Pancreatic Surgery [19], ISGPS type IBS0) for hard stumps and duct-to-mucosa with or without externalized stents (ISGPS type IAS0 and IAS2) or pancreaticogastrostomy (ISGPS type II) for soft pancreatic stumps. Both pylorus-preserving and Whipple procedures were included in the study. Minimally invasive procedures were not included. Postoperative course was managed by a team of surgeons composed by residents of all postgraduate years under the constant supervision of attending surgeons.

POPF was defined according to the updated ISGPS definition [20] that was retrospectively applied to patients treated before 2016. Additionally, postpancreatectomy hemorrhage (PPH) and delayed gastric emptying (DGE) were defined according to ISGPS definitions [21, 22]. Thirty-day postoperative morbidity was rated according to the Clavien-Dindo classification [23]. The failure-to-rescue rate was calculated as 90-day mortality (numerator) among patients experiencing severe morbidity (denominator), defined as Clavien-Dindo ≥ 3. The burden of the surgical procedure and preoperative patients’ health status was expressed through the concept of benchmarking. A “benchmark case” was defined as a standard Whipple procedure in a surgically fit patient in which the best achievable results are expected. Specific criteria to define a benchmark case have been recently identified in a multicentric international study [24].

The primary endpoint was the incidence of Clavien-Dindo ≥ 3 morbidity that was reappraised according to the month in which the procedure was performed to identify possible seasonal variations. Three different hypotheses were assessed. First, the effect of climate variations, expressed by maximum and minimum average temperature measured in Celsius degrees registered in the city of Verona and reported in regional registries [25].

Second, the effect of the introduction of new post-graduate year 1 residents in the clinical and surgical activity. Since the introduction of new trainees took place every year in a different month—both in the first and in the second semester of the year—according to the regulations of the Italian Ministry of Education, University and Research, we assessed the possible “July effect”, as reported by the North American literature [2–4], by comparing the outcomes of patients operated during the first three months of post-graduate year 1 to those of patients operated in the remaining 9 months.

Third, the effect of staff vacation by comparing outcomes of patients operated during months at higher rates of sabbaticals vs. rest of the year. In Italy, the months of July, August and September are the most used for summer vacations, whereas December is characterized by the Christmas holidays. These months were considered as months with a higher percentage of staff on vacation. Data about staff on vacation per month of the year were retrieved by hospital registries focusing on medical and nursing staff of the department of surgery, medical staff of the department of diagnostics and of the Intensive Care Unit.

Once a specific pattern was identified, further analyses were performed on patient characteristics and specific surgical outcomes. Eventually, we explored whether being operated on during a specific time of the year could be a predictor of severe morbidity or increased mortality.

### Statistical analysis

Continuous variables are presented as the median and interquartile range (IQR). Dichotomous variables were presented as frequencies and proportions. Differences between groups were tested using the Mann–Whitney *U* test for numeric variables and the chi-square test or Fisher’s exact test for dichotomous variables. Stepwise backward logistic regression analysis was used to identify covariates associated with the incidence of Clavien-Dindo ≥ 3 morbidity. All tests were two-tailed. *P* values < 0.05 were considered statistically significant. Statistical analysis was performed with SPSS software (SPSS Inc., version 20 for Macintosh, IBM, Chicago, Il).

## Results

A total of 2748 Whipple procedures were included in the present study. The overall rates of 90-day Clavien-Dindo ≥ 3 morbidity and 90-day mortality were 19.7 and 2%, respectively. The difference in the severe morbidity rate between months was statistically significant (*p* = 0.047), but the difference in mortality rates stratified by month was not statistically significant (*p* = 0.940).

**Fig. 1:**
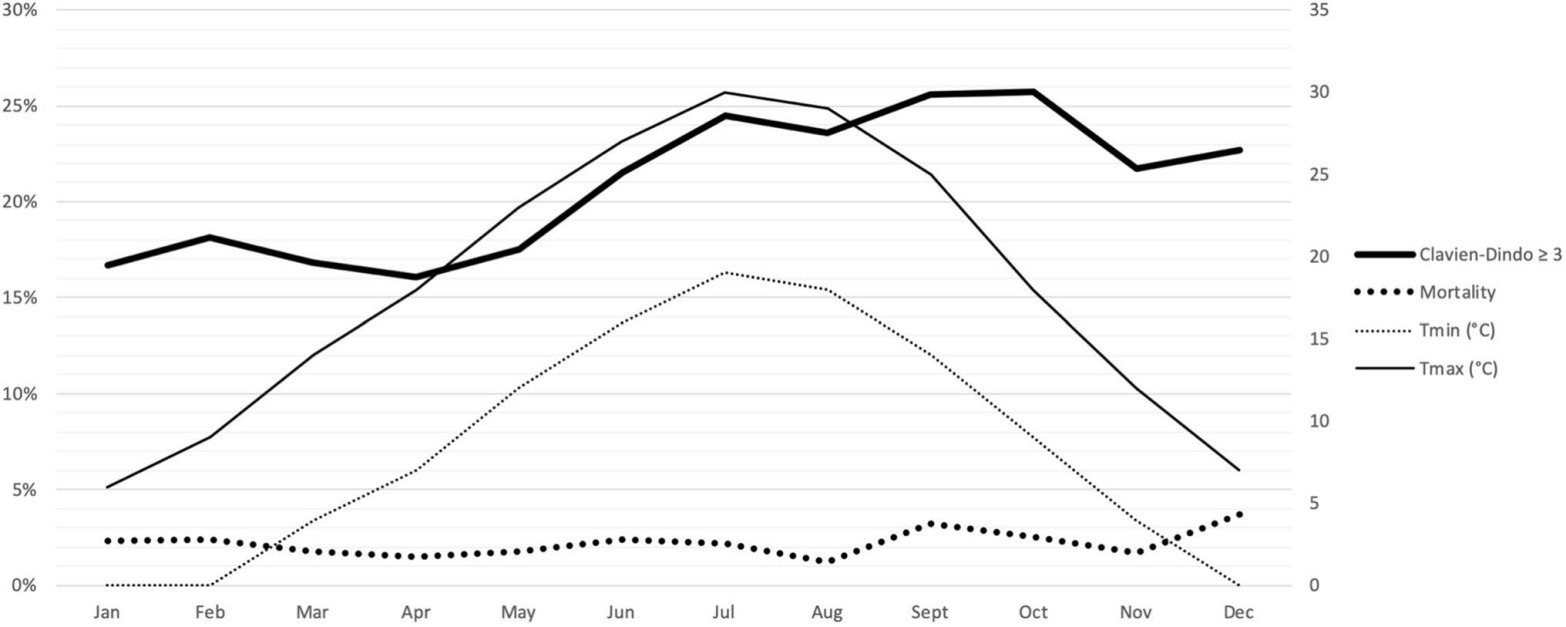
Relationship between Clavien-Dindo ≥ 3 morbidity and mortality rates (y-axis left) and maximum and minimum temperature (y-axis right) according to the month of the surgical procedure (study period 2000–2018)

Figure 1 shows a graphical analysis of severe morbidity and mortality rates with the average minimum and maximum temperature each month over a 19-year period. There was only a partial overlap of the pattern of the incidence of severe morbidity and mortality with the seasonal increase in the average maximum and minimum temperature, which, instead, was prominent only between May and September. Comparing patients operated during warm months (from May to September) to those operated during cold months (from October to April) there were no differences in terms of Clavien-Dindo ≥ 3 morbidity (22.5% vs 19.8%, *p* = 0.104) and 90-day mortality (2.2% vs. 2.2%, *p* = 1.000). Focusing on surgical site infections, that usually increase during warmer months, there was no difference in terms of wound infection comparing warm to cold months (6.1 vs. 5.2%, *p* = 0.340).

With respect to the role of the “July effect”, data were not plotted since the introduction of new trainees varied through the study period. There was no difference in terms of Clavien-Dindo ≥ 3 morbidity (23.5% vs 22.5%, *p* = 0.757) and 90-day mortality (3.8% vs. 3.2%, *p* = 0.289) when patients operated during the first 3 months of a new class of post-graduate year 1 trainees were compared to patients operated during the rest of the year.

**Fig. 2:**
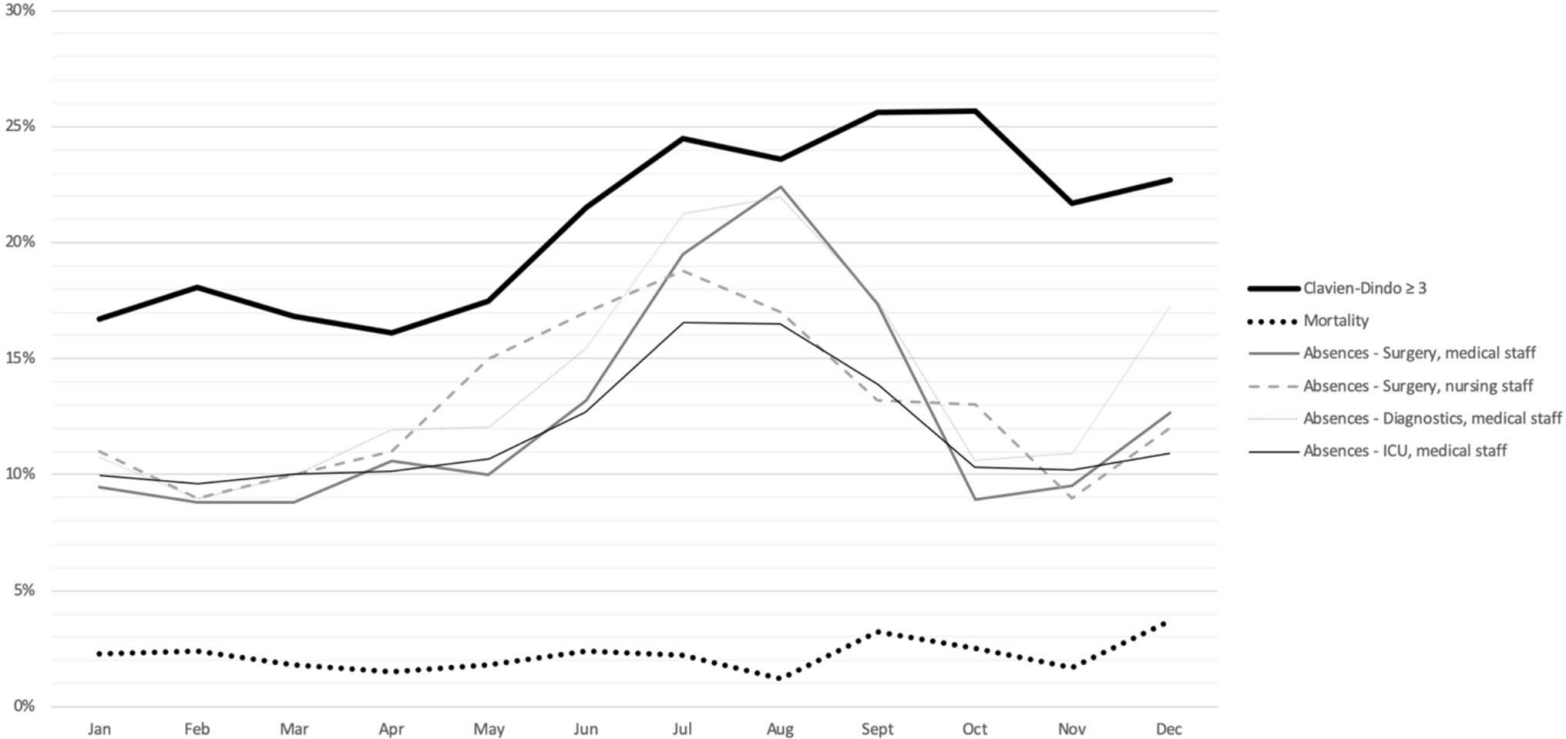
Relationship between Clavien-Dindo ≥ 3 morbidity and mortality rates and percentage of staff members on vacation (for the Department of Surgery, Department of Diagnostics and ICU) according to the month of the surgical procedure (study period 2000–2018)

Figure 2 shows the relationship between the seasonal variability of severe morbidity and mortality and the percentage of staff members on vacation each month over a 19-year period. The percentage of faculty on vacation was almost constant during the months from January to June and during the months of October and November, whereas it almost doubled during the summer months of July, August and September and on December. Quite similar rates of staff on vacation were observed for the nursing staff of the Department of Surgery and for the medical staff of the Department of Diagnostics. Interestingly, the amount of Intensive Care Unit staff on vacation during the summer months and on December was less marked. To allow for the staff turnover during summer months, the activity of the entire Department is partially diminished. For this reason, fewer beds and operating theaters are available for elective surgery especially on July and August. Indeed, the monthly caseload reached the nadir on August with a median number of 13 cases considering the entire study period. At the same time, the personnel shortage in the Department of Diagnostics increases the waiting list for access to the necessary preoperative work-up. All these changes involve a cyclic increase in the surgical waiting list during the summer months. Indeed, considering only Whipple procedures scheduled for upfront resection for pancreatic ductal adenocarcinoma, surgical waiting list was significantly longer for patients operated during summer months (median time 37 vs. 27 days, *p* = 0.037).

Given these results, the hypothesis of a detrimental effect on surgical outcomes produced by a higher percentage of staff on vacation was further explored. Patients were then divided into two groups: those operated on July, August, September and December (*n* = 834), when the amount of staff on vacation is higher, and those operated during the rest of the year (*n* = 1914).

Table 1 shows the baseline characteristics of the two populations. Patients who underwent surgery during the months of July, August, September and December have only a significantly increased incidence of preoperative weight loss.

**Table 1:** Baseline characteristics of patients undergoing PD during months with a low vs. high percentage of staff on vacation

	Percentage of staff on vacation		*p*
	Low (*n* = 1914)	High (*n* = 834)	
Sex			
M	1105 (57.7%)	469 (56.2%)	0.476
F	809 (42.3%)	365 (43.8%)	
Age (median, IQR)	64 (15)	64 (16)	0.992
BMI (median, IQR)	24 (4.3)	24.2 (4.6)	0.201
Neoadjuvant therapy	222 (11.5%)	118 (14.1%)	0.067
ASA			
I	70 (5.9%)	58 (5%)	0.568
II	889 (74.8%)	886 (76.4%)	
III	227 (19.1%)	211 (18.2%)	
IV	2 (0.2%)	4 (0.3%)	
Benchmark case	1192 (62.2%)	512 (61.4%)	0.669
Weight loss	661 (34.5%)	326 (39%)	0.025

Table 2 compares data on surgical outcomes during the two periods of the year. There was no difference in terms of incidence of POPF, PPH or DGE, but being operated during months with a higher percentage of staff on vacation was associated with a significantly increased incidence of abdominal abscesses, sepsis and Clavien-Dindo ≥ 3 morbidity.

**Table 2:** Surgical outcome after PD comparing months with a low vs. high percentage of staff on vacation

	Percentage of staff on vacation		*p*
	Low (*n* = 1914)	High (*n* = 834)	
POPF	351 (18.3%)	148 (17.7%)	0.747
B	299 (16.6%)	123 (16.5%)	0.900
C	52 (2.9%)	25 (3.4%)	
PPH	218 (11.4%)	99 (11.9%)	0.745
A	42 (2.2%)	13 (1.5%)	0.209
B	105 (5.5%)	56 (6.7%)	
C	71 (3.7%)	30 (3.6%)	
DGE	191 (9.9%)	84 (10%)	0.945
A	43 (2.2%)	10 (1.2%)	0.333
B	107 (5.6%)	57 (6.8%)	
C	41 (2.1%)	17 (2%)	
Abscess	299 (15.6%)	157 (18.8%)	0.039
Wound infection	92 (5.3%)	43 (5.9%)	0.561
Pneumonia	372 (19.4%)	190 (22.8%)	0.051
Cardiac morbidity	30 (1.5%)	16 (1.9%)	0.520
Acute renal failure	32 (1.6%)	20 (2.4%)	0.223
Sepsis	154 (8%)	90 (10.7%)	0.024
Relaparotomy	121 (6.3%)	50 (6%)	0.797
Clavien-Dindo ≥ 3	354 (18.5%)	156 (22.3%)	0.022
Mortality	36 (1.8%)	19 (2.3%)	0.553

Table 3 reports univariate and multivariable analyses of predictors of severe complications. Together with male sex and weight loss, being operated during months with a higher percentage of staff on vacation was confirmed as an independent predictor of severe morbidity after Whipple procedure.

**Table 3:** Univariate and Multivariable analysis of predictors of severe morbidity (Clavien-Dindo ≥ 3) after PD

	Clavien-Dindo ≥ 3		*p*	OR	CI 95%	*p*
	No (*n* = 2043)	Yes (*n* = 540)				
Sex (male)	1120 (54.8%)	339 (62.8%)	0.001	1.530	1.194 – 1.962	**0.001**
Age (median, IQR)	62 (16)	65 (15)	0.026	1.09	0.998 – 1.021	0.125
BMI (mean, SD)	24.3 (3.7)	24.9 (3.4)	< 0.001	1.033	0.999 – 1.068	0.060
Neoadjuvant therapy	273 (13.8%)	64 (12.2%)	0.388			
ASA score ≥ 3	322 (17.4%)	120 (25.2%)	< 0.001	1.088	0.676 – 1.750	0.728
Surgery during months with a high percentage of staff on vacation	608 (29.8%)	194 (35.9%)	0.007	1.271	1.086 – 1.638	**0.031**
Weight loss	769 (48%)	171 (40.8%)	0.008	1.495	1.170 – 1.910	**0.001**

## Discussion

The present work shows how seasonal variation in surgical outcomes is a real and measurable phenomenon using a high-volume pancreas center as a model. While the mortality rate does not show a specific pattern, severe morbidity rates significantly vary during the year. This phenomenon does not appear to be related to seasonal climate changes, and it is not affected by the access of new and inexperienced trainees to patient care. However, undergoing a Whipple procedure during the peak of provider vacations represents an independent predictor of severe morbidity. Such an increased severe morbidity rate during the months where staff vacations are concentrated is certainly a multifactorial event that cannot be explained by a single hypothesis. Moreover, each specific hypothesis is difficult to demonstrate since environmental or socioeconomic factors are possibly involved.

Studies on outpatient clinic populations have demonstrated increased all-cause mortality during the winter months, as the cold weather can lead to several alterations that increase mortality due to respiratory and cardiovascular diseases [26, 27]. However, since the community environment is extremely different, this evidence cannot be generalized to a cohort of hospitalized patients. Previous reports have shown how warmer temperatures and humidity can facilitate bacterial colonization in the nosocomial environment, leading to a higher risk of surgical site infection [9]. In the present study, we did not identify an increased rate of surgical site infections during the warmer months. Moreover, when the variations in the temperature and severe morbidity rate were compared, the two identified patterns did not correlate. These results could be explained by the fact that modern hospital facilities enable accurate control of air temperature and humidification levels, so that these are not affected by seasonal variations.

Another specific cause that has been addressed in previously published studies is the disruption of the complex hospital system caused by the influx of new trainees in surgical wards, operating theaters and intensive care units. As they gain access to patient care, their inexperience could be the cause of the increased severe morbidity rate that reaches its maximum during the month in which the academic year begins and then progressively decreases due to the accumulation of experience. Such “July effect”, as the introduction of new residents in the United States system usually take place on July, has been reported by several papers, but evidence of its actual impact on postoperative outcomes is controversial [2–4, 28, 29]. This analysis was performed to compare the Italian system with the North American one to assess the possible influence of new trainees on surgical outcomes. As expected, major morbidity and mortality are not increased when new residents are introduced in the surgical ward and in the operating theater. Unlike the North American system, new residents began their activity in different periods in Italy, both in the first and second half of the year. Moreover, at our center, new residents are constantly tutored by consultants or senior residents and are only progressively involved in tasks with greater responsibility.

A third mechanism that can explain seasonal variations in surgical outcomes is staff and resource shortage during vacation periods. Several studies have demonstrated worse outcomes for procedures performed on Fridays [10], on weekends [11] or during vacation or holiday periods [12], regardless of the specific month in which they take place worldwide [30, 31]. During holidays, hospital systems are significantly disrupted, as significant segments of the staff are off work and care capacity is usually delivered by less experienced/occasional staff and with less overall resource availability. Although these phenomena are not easily measurable in the surgical department, they represent evidence for anyone working in an academic environment, particularly in facilities of the national health system. Because of the mild and temperate climate, the summer vacations in Italy take place mainly between July and September [32]. Many people also choose the month of December to plan their vacation to spend time with family during Christmas time. Due to personnel shortage and to allow staff turnover during the summer period, the hospital services are partially reduced especially on July and August. In details, fewer hospital beds are available, fewer radiological examinations can be scheduled and fewer operating theaters can be used. This eventually leads to an extension of the surgical waiting list and to a less prompt and effective preoperative work-up. This is of particular impact in the field of pancreatic surgery, as patients often require preoperative jaundice palliation, nutritional support, and more than one cross-sectional imaging examination or other endoscopic procedure. Because of the length of the surgical waiting list, the detrimental effect of summer vacations on preoperative management may continue for several weeks. This might explain why the detrimental effect of holidays on the rate of severe morbidity extends up to October. Interestingly, the increased rate of severe morbidity did not match with an increased mortality rate, probably because the ICU staff and the experience of the professional employees at the center were not significantly affected by resource shortages during summer vacations. This evidence is supported by the fact that ICU staff shortages are less evident during the summer months than that of Surgery or Diagnostics (Fig. 2). Although it is difficult to identify a cause-effect relationship, this paper is in line with multiple others on the same topic assuming that specific cultural and environmental factors (e.g., vacations) might play a major role in medical and surgical outcomes [10–12].

This study has several limitations. Aside from the inherent drawbacks of using a large prospectively collected database with limited data, we were unable to control for particular variables that may have influenced outcomes after a major pancreatic resection. Subtle changes of practice including new faculty, accuracy of data collection, the introduction of standardization of outcome metrics, standardized clinical pathways for an enhanced recovery, and the extension of surgical indications due to the large use of neoadjuvant therapy may have played an important role. Moreover, this is a single-center Italian study that allows for a high level of standardization of patient management over the years but probably hampers external validity in other countries, especially in private healthcare settings. Results coming from the analysis involving climate changes cannot be exported in other countries outside the Mediterranean area. Seasonal variation of surgical outcomes should be interpreted as a multifactorial issue and it is complex to identify a definite cause-and-effect mechanism. However, this analysis provides an overview that allows to identify some critical areas in which action is required, such as resource shortage during periods of vacation.

## Conclusion

Seasonal variability in surgical outcomes after the Whipple procedure is a real and measurable phenomenon at a high-volume academic center of the national healthcare system. The severe morbidity rate is significantly increased during months with more staff on vacation, whereas mortality remains constant. This evidence does not seem to be linked to seasonal climate change or to the arrival of new and inexperienced residents but rather to resource shortages during these months. The organization of services during vacation periods should be improved to guarantee the continuation of high-level care.
